# Naphthalene Diimide and Pyromellitic Diimide Networks as Cathode Materials in Lithium‐ion Batteries: on the Instability of Pyromellitic Diimide

**DOI:** 10.1002/marc.202401121

**Published:** 2025-01-13

**Authors:** Rukiya Matsidik, Daniele Fazzi, Andreas Seifert, Michael Sommer

**Affiliations:** ^1^ Institut für Chemie Technische Universität Chemnitz Straße der Nationen 62 09111 Chemnitz Germany; ^2^ Forschungszentrum MAIN Technische Universität Chemnitz Rosenbergstraße 6 09126 Chemnitz Germany; ^3^ Department of Chemistry “Giacomo Ciamician” University of Bologna via P. Gobetti 85 Bologna 40129 Italy

**Keywords:** cationic polymerization, naphthalene diimide, organic battery, polymer cathode, pyromellitic diimide

## Abstract

Aromatic diimides such as naphthalene diimide (NDI) and pyromellitic diimide (MDI) are important building blocks for organic electrode materials. They feature a two‐electron redox mechanism that allows for energy storage. Due to the smaller size of MDI compared to NDI its theoretical capacity is higher. Studies on MDI‐based small molecule and linear polymer electrodes indicate that MDI is unstable, yet the origin of instability remains unclear. Herein, two cross‐linked networks of NDI and MDI are designed. The polymers, termed PNDI‐EG and PMDI‐EG, are synthesized via cationic polymerization of vinyl ethylene glycol‐functionalized NDI and MDI monomers. The cross‐linked structures preclude extrinsic degradation pathways (e.g., dissolution in the electrolyte), and thereby facilitate the investigation of intrinsic degradation mechanisms. PMDI‐EG‐based cathodes are less stable, and the performance of PMDI‐EG/Li half cells is markedly inferior compared to PNDI‐EG/Li cells. Our comprehensive experimental and quantum‐chemical investigation reveals that PMDI‐EG undergoes irreversible diimide ring opening upon prolonged charge–discharge cycles, while PNDI‐EG remains intact. It is hypothesized that the smaller ring size of the five‐membered imide renders MDI more susceptible to side reactions with nucleophiles in the electrolyte, causing rapid loss of capacity during the first cycles.

## Graphical abstract

Crosslinked polymers of napthalene and pyromellitic diimide, functionalised with ethylene glycol chains (PMDI‐EG and PNDI‐EG) are synthesized in two steps combining Mitsunobu reaction and cationic polymerization. PMDI‐EG cathodes undergo irreversible changes upon prolonged battery cycling, whereas PNDI‐EG remain intact. Imide ring opening for MDI is identified as degradation reaction, possibly caused by a larger ring strain as compared to NDI.

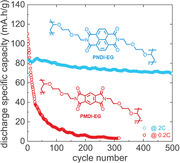

## 1 Introduction

In the search for sustainable alternative materials for emerging battery technologies, organic electrode materials (OEMs) have recently gained significant renewed interest.^[^ ^1^, ^2^, ^3^, ^4^, ^5^ ^]^ OEMs are composed of abundant elements such as C, H, N, O, and S, and their structure can be tailored to meet desired properties. The inherent processability of OEMs allows for energy efficient processing of the electrodes, while the absence of metals facilitates an end‐of‐life disposal.^[^ ^1^, ^3^, ^4^ ^]^ Most intriguingly, electrical energy storage in OEMs functions though fast and, ideally, reversible redox reactions. OEMs can be made electroactive with other, more abundant metals beyond lithium,^[^ ^6^ ^]^ such as sodium,^[^ ^7^ ^]^ magnesium,^[^ ^8^ ^]^ aluminum,^[^ ^9^ ^]^ and zinc.^[^ ^10^ ^]^ Moreover, they can be combined with air and with other OEMs.^[^ ^5^ ^]^ OEMs have therefore the potential to provide cost‐effective energy storage solutions while stimulating the development of novel battery concepts.

Among the various types of OEMs, organic carbonyl compounds (OCCs) are an important class.^[^ ^11^, ^12^, ^13^ ^]^ In OCCs designed for energy storage, the oxygen of the carbonyl group is reduced upon reduction of the OCC. The resulting negative charge must be stabilized with, e.g., aromatic rings.^[^ ^11^ ^]^ Aromatic diimides such as pyromellitic diimide (MDI) and naphthalene diimide (NDI) are the two structurally most simple examples of OCCs that feature a reversible two‐electron redox mechanism. Importantly, substitution at the aromatic core and the imide nitrogen allow for efficient tuning of their structure,^[^ ^14^, ^15^ ^]^ incorporation into polymer architectures^[^ ^16^, ^17^, ^18^, ^19^, ^20^ ^]^ and covalent organic frameworks (COFs).^[^ ^21^, ^22^ ^]^ MDI and NDI have thus become versatile building blocks for OEMs. Due to its smaller size, MDI has a significantly higher theoretical capacity of 248 mA•h g^−1^ compared to NDI (201 mA•h g^−1^) or other larger aromatic diimides such as perylene diimide (PDI, 137 mA•h g^−1^).^[^ ^23^ ^]^ The structural simplicity and high theoretical capacity make MDI an attractive motif for OEMs.

Several studies using small‐molecule and linear polymers have shown that MDI exhibits a considerably inferior cycling stability in comparison to NDI and PDI.^[^ ^23^, ^24^, ^25^, ^26^, ^27^ ^]^ Indeed, the larger the size of the stabilizing aromatic rings on OCCs, the greater the stabilization of the negatively charged carbonyl groups.^[^ ^11^, ^28^ ^]^ However, this increased stabilization comes at the cost of specific capacity, as molecular weight increases while the two‐electron redoxation remains the same.^[^ ^11^, ^23^ ^]^ Based on electron paramagnetic resonance (EPR) measurements and density functional theory (DFT)‐based calculations, Chen et. al. concluded that the radical anion intermediate formed after one‐electron reduction is less stable in case of MDI due to a rather localized spin density.^[^ ^23^ ^]^ Others have noted that irreversible processes and decompositions occur in MDI electrodes, as evidenced by post‐mortem nuclear magnetic resonance (NMR) spectroscopy experiments.^[^ ^24^, ^26^ ^]^ However, the origin of these degradation pathways remains insufficiently explored. Moreover, all of the aforementioned studies have been conducted on either small molecules or linear polymers of MDI. This raises the possibility that other degradation mechanisms, such as material dissolution into the electrolyte, cannot be excluded, especially as MDIs exhibit generally higher solubility than NDIs and PDIs. The cycling performance of MDI‐based COFs has been reported to be highly stable.^[^ ^21^, ^29^ ^]^ However, other factors, such as porosity and nanostructure, also play a crucial role in this regard. The multitude of factors at play therefore make it challenging to pin down molecular parameters that are responsible for the inferior cycling stability of MDIs in comparison to larger analogs.

**Scheme 1:**
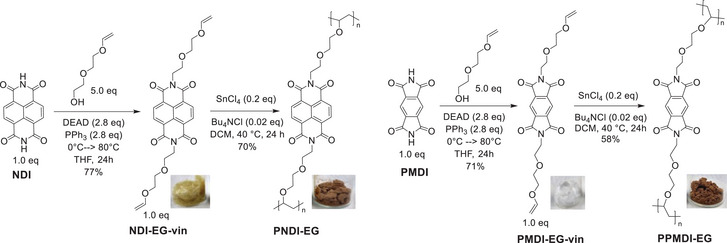
Synthetic routes and isolated yields of monomers (NDI‐EG‐vin and MDI‐EG‐vin) via Mitsunobu reaction and cross‐linked‐polymers (PNDI‐EG and PMDI‐EG) via cationic polymerization. DEAD: diethyl diazenedicarboxylate; PPh_3_: triphenylphosphine; THF: tetrahydrofuran; DCM: dichloromethane.

To address this issue, we designed two simple MDI and NDI monomers MDI‐EG‐vin and MDI‐EG‐vin, that are symmetrically functionalized with vinyl (vin) functionalities and ethylene glycol (EG) linkers at the imide position (**Scheme** **1**). These monomers were polymerized via cationic polymerization to obtain cross‐linked, amorphous and insoluble polymers termed PMDI‐EG and PNDI‐EG (Scheme 1; Figure S1, Supporting Information). This design strategy allows for the investigation of the differences between MDI and NDI, eliminating potential differences in solubility or microstructure. Under identical electrode composition, structure, and testing conditions, PNDI‐EG/Li half cells exhibited markedly superior cycling stability and rate capability compared to PMDI‐EG/Li half cells. Fourier‐Transform Infrared (FT‐IR) spectroscopy and solid‐state NMR (ssNMR) analysis of post‐mortem cathodes indicate that during prolonged charge–discharge cycles, the five‐membered imide ring of MDI degrades, leading to imide ring opening and loss of capacity. Such degradation is not found in PNDI‐EG. While these findings deepen the understanding of MDI‐based OMEs, the simple linker chemistry may be useful for the preparation of other polymeric electrode materials.

## 2 Results and Discussion

### 2.1 Synthesis and Characterization

MDI‐EG‐vin and NDI‐EG‐vin monomers were synthesized in a single step using the Mitsunobu reaction of pyromellitic diimide (MDI) and 1,4,5,8‐naphthalenediimide (NDI) with commercially available and inexpensive diethylene glycol monovinyl ether in good yields (Scheme 1). Purification of the monomers was also straightforward by first filtering through a short silica plug followed by recrystallization as described in the **Experimental Section**. The vinyl ether in NDI‐EG‐vin and MDI‐EG‐vin do not allow for free‐radical polymerization. Inverse vulcanization recently developed for propenyl‐substituted NDI^[^ ^30^, ^31^ ^]^ was unsuccessful due to the observed low reactivity, particularly of MDI‐EG‐vin. Instead, cationic polymerization with SnCl_4_ and a catalytic amount of Bu_4_NCl in DCM was found to be effective for both NDI‐EG‐vin and MDI‐EG‐vin monomers furnishing PNDI‐EG and PMDI‐EG, respectively.

**Figure 1:**
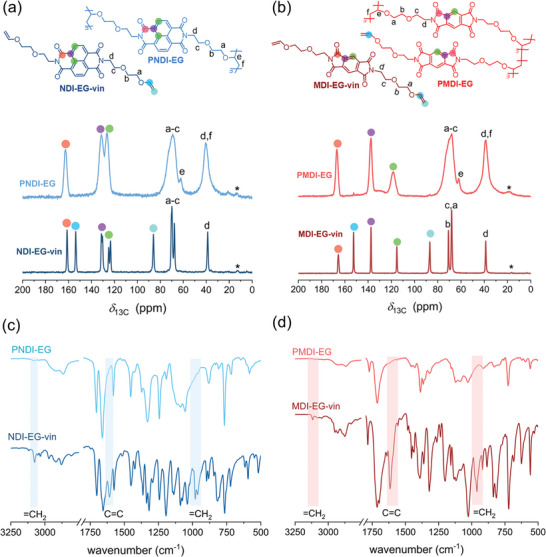
(a, b) ^13^C ssNMR spectra of monomers (dark color) and polymers (light color) in solid state, * denotes a spinning side band. (c, d) FT‐IR spectra of monomers (dark color) and polymers (light color).

Due to their cross‐linked structure both polymers are insoluble in common organic solvents, including the electrolyte solvents 1, 2‐dimethoxy‐ethane (DME) and 1,3‐dioxolane (DOL) (Figure S1, Supporting Information). Therefore, we applied solid state methods to characterize the polymers. The ^13^C ssNMR analysis of the cross‐linked polymers (**Figure** **1**a,b) revealed the absence of signals from the vinyl group carbons in comparison to the monomers (Figure 1, cyan and blue colors), indicating full monomer conversion during cationic polymerization. The comparison of the FT‐IR spectra of monomers and polymers further confirm full monomer conversion and the successful preparation of cross‐linked network structures. The distinct stretching and bending vibrations of the terminal vinyl groups^[^ ^32^ ^]^ of the monomers, indicated by blue (NDI‐EG‐vin) or red (MDI‐EG‐vin) shades in Figure 1c,d, are absent in the polymers (Figure 1c,d). Both PMDI‐EG and PNDI‐EG exhibit a high thermal stability with similar onset temperatures at ≈360 °C probed by thermogravimetric analysis (Figure S2, Supporting Information). The smaller aromatic core in MDI results in a lower glass transition temperature (*T*_g_) for PMDI‐EG (130 °C) compared the *T*_g_ of PNDI‐EG (160 °C) (Figure S2, Supporting Information).

### 2.2 Electrochemical Characterization of PMDI‐EG and PNDI‐EG Cathodes

**Figure 2:**
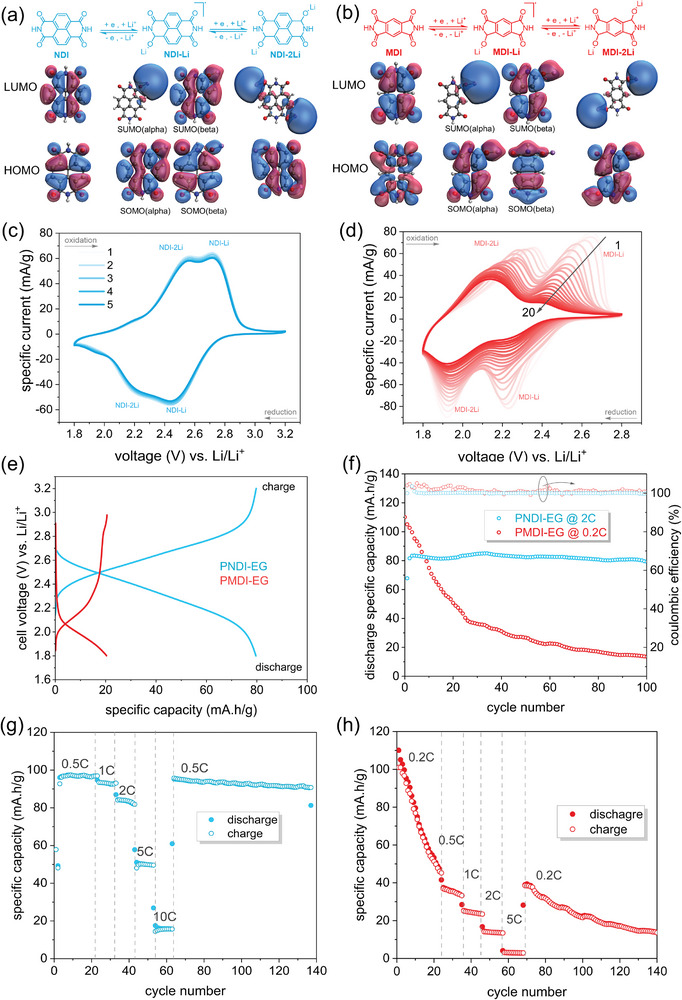
The reversible two‐electron reduction of NDI (a) and MDI (b) and DFT calculated frontiers molecular orbitals of the neutral and lithiated species formed. c,d) CV curves with a scan rate of 0.1 mV s^−1^, e) characteristic charge–discharge voltage profile, f) discharge specific capacity and columbic efficiency from long‐term charge–discharge cycling, and g,h) rate capability tests of PNDI‐EG (blue curves) and PMDI‐EG (red curves) composite cathodes in a half cell configuration.

Electrochemical characteristics and battery performance of PMDI‐EG and PNDI‐EG cathode materials were investigated using a composite electrode formulation containing 76 wt.% active polymer, 19 wt.% multiwalled carbon nanotubes (MWCNT) and 5 wt.% polyvinylidene difluoride (PVDF), as detailed in the Experimental Section. These composite cathodes were paired with Li‐anodes to form half cells. Cyclic voltammetry (CV) tests were first performed to gain insight into the redox behavior of the cathodes. Furthermore, the molecular orbitals (MO) and MO energy levels of the various species formed during the two‐electron redox process were calculated for the simplified NDI and MDI motifs having N‐H termination at the DFT (B3LYP‐D3/cc‐pVTZ) level of theory (Table S1, Supporting Information; **Figure** **2**a,b). All the highest occupied MOs (HOMO), for both the pristine and the lithiated species, are delocalized over the NDI and MDI motifs and exhibited π‐character. The only exception is the MDI species in the pristine case, where the lone pairs of the oxygen atoms couple with the σ‐orbitals of the backbone. The lowest unoccupied MOs (LUMO) of the pristine species are delocalized as well on the molecular backbone both in case of NDI and MDI. However, when one or two lithium are interacting with the oxygen atoms, the LUMO becomes strongly localized over the lithium centers. For the pristine species the HOMO of MDI is deeper in energy than that of NDI (−8.02 eV vs −7.43 eV, Table S1, Supporting Information). The LUMO level of NDI (−3.81 eV) is more negative than that of MDI (−3.54 eV), indicating a higher stability of NDI upon reduction. This trend is confirmed by the HOMO energy levels of the mono‐ and di‐lithiated species, where MDI (mono‐Li (−4.13 eV), di‐Li (−3.08 eV)) exhibits more positive values compared to NDI (mono‐Li (−4.29 eV), di‐Li (−3.68 eV)), and therefore can be assumed to be less stable than NDI upon lithiation (see MO energies as reported in Table S1, Supporting Information).

The CV curves indicate that PNDI‐EG undergoes reductions at higher potentials (vs Li/Li^+^) than PMDI‐EG. The first and second reductions, which result in the formation of one and two lithiated species (NDI‐Li/MDI‐Li and NDI‐2Li/MDI‐2Li), as illustrated in Figure 2a,b, give rise to two reduction peaks at 2.45 V/ 2.23 V in PNDI‐EG (Figure 2c) and at 2.23 V/1.92 V in PMDI‐EG (Figure 2d), respectively. This indicates a lower LUMO of PNDI‐EG compared to PMDI‐EG, which aligns well with the DFT‐calculated MO energy levels (Tables S1, Supporting Information). The corresponding two oxidation peaks were observed at 2.72 V/2.55 V for PNDI‐EG and at 2.65 V/2.34 V for PMDI‐EG. Consequently, the two‐electron redox process is separated by 0.27 V/ 0.32 V for PNDI‐EG and 0.42 V /0.42 V for PMDI‐EG, indicating slower reaction kinetics for PMDI‐EG.^[^ ^33^ ^]^ For PNDI‐EG cathodes, this reversible two‐electron redox process is highly stable, as evidenced by the similar specific current and peak positions observed in multiple CV scans (Figure 2c). However, for the PMDI‐EG composite cathodes the two‐electron redox process undergoes a significant deterioration upon multiple CV cycles, resulting in a reduction in specific current with each CV cycle (Figure 2d). From the differences in the specific current derived from the first and 20th CV cycles, the first redox peak at 2.23 V/2.66 V decays faster than the second one. In addition to the reduction in peak current during CV cycles, the peak position of the oxidation reactions also shifts to lower potentials, indicating an increase in HOMO energy levels.

For NDI‐based cathodes, the herein applied electrolyte formulation (lithium trifluoromethane sulfonimide (LiTSFI) in DME: DOL mixture) has been shown to give the most stable performance.^[^ ^28^ ^]^ To the best of our knowledge, the effect of electrolyte on the performance of MDI‐based electrodes has yet to be investigated. PMDI‐EG cathodes were also tested with other electrolyte formulations such as LiTSFI in dimethyl carbonate (DMC) or lithium hexafluorophosphate (LiPF_6_) in DMC. Under all these conditions, PMDI‐EG cathodes again exhibited poor cycling stability (Figure S3, Supporting Information). For further studies, we therefore maintained the original electrolyte formulation (LiTSFI in DOL: DME) for both PMDI‐EG and PNDI‐EG.

We next investigated battery performance of the composite cathodes. Based on the redoxation peak positions in the CV curves, cutoff voltages were set to 1.8–3.2 V for PNDI‐EG and 1.8–3.0 V for PMDI‐EG. From the voltage profiles of the half cells (Figure 2e), the PMDI‐EG composite cathodes exhibit a markedly lower cell potential of 2.1 V, which is 0.4 V less than the PNDI‐EG composite cathodes (2.5 V). When cycled at 0.2 C, the PMDI‐EG based cathodes deliver a specific discharge capacity of 110 mA.h g^−1^ in the first cycle, which is 90% of its theoretical capacity (122 mA•h g^−1^). This is slightly higher compared to the PNDI‐EG cathode which delivers 97 mA•h g^−1^ (88% of its theoretical capacity 110 mA•h g^−1^) at 0.5 C. However, the capacity of PMDI‐EG declines rapidly with cycle number, losing 91% of its initial capacity after 100 charge–discharge cycles (Figure 2f, red line). This corresponds to ≈1% capacity loss per cycle. Despite poor cycling stability, PMDI‐EG composite cathodes show excellent columbic efficiency (CE) reaching over 100%. In comparison, PNDI‐EG composite cathodes exhibit both excellent cycling stability and CE, and retain 96% of initial capacity after 100 cycles (0.04% loss per cycle) (Figure 2f, blue line). When cycled at a much higher rate of 2C, PNDI‐EG cathodes retain 81% of initial capacity after 1000 cycles (0.02% loss per cycle) (Figure S4, Supporting Information). Due to their instability combined with presumably slow reaction kinetics, PMDI‐EG composite cathodes also showed poor rate capability (Figure 2g). In contrast, the PNDI‐EG composite cathodes again demonstrate significantly superior rate capability (Figure 2h). At a rate of 0.5 C, the specific discharge capacity was observed to be 97 mA•h g^−1^. Upon increasing the C‐rate to 1 and 2 C, the capacities exhibited slight decreases, reaching 93 and 84 mA•h g^−1^, respectively. At 5 C, a markedly diminished capacity of 50 mA•h g^−1^ was observed, which exhibited a further decline to 10 mA•h g^−1^ at 10 C. Notably, upon subsequent cycling at a low C‐rate of 0.5 C, the capacity recovered to over 90 mA•h g^−1^.

### 2.3 Origin of Degradation

**Figure 3:**
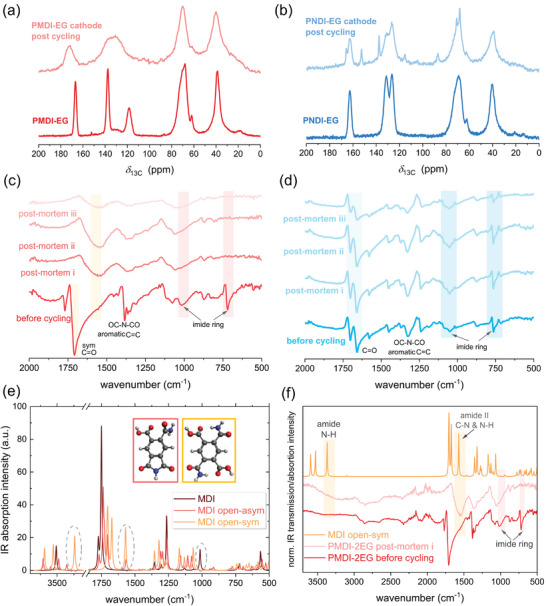
^13^C ssNMR spectra (a, b) and IR spectra (c, d) and of PMDI‐EG (a, c) and PNDI‐EG (b, d) composite cathodes before cycling (dark color) and postmortem (light color) (i: after 300 cycles (PMDI‐EG at 0.2C) and 1000 cycles (PNDI‐EG at 2C), ii) after 20 CV cycles, iii: after 150 cycles at different C‐rates shown in Figure 2g,h) cycles). DFT calculated IR spectra of natural, asymmetrically, and symmetrically ring opened MDI (e), and IR spectra comparison of PMDI‐EG cathode and post‐mortem cathode to the simulated symmetrically ring‐opened MDI (f).

In order to gain a deeper understanding of the origin of degradation, post‐mortem composite cathodes were subjected to characterization by ssNMR and FT‐IR spectroscopy. FT‐IR spectra of the various species formed during the two‐electron redox process were also examined with the aid of DFT calculations. In the ^13^C ssNMR spectrum of the post‐mortem PMDI‐EG cathode (**Figure** **3**a), the peak positions shifted to lower field compared to the pristine MDI network, while the peak at 119 ppm arising from the unsubstituted aromatic carbon of MDI core seemed to be absent. Moreover, the signals of the MDI core between 110 and 180 ppm became significantly broader and less intense, indicating the formation of new species with carbonyl and aromatic groups at the cost of the MDI core upon charging and discharging. In contrast, the ^13^C ssNMR characteristics of post‐mortem PNDI‐EG cathodes remained largely unaltered, giving peaks that have similar chemical shifts and intensities compared to the spectrum of pristine PNDI‐EG (Figure 3b). In addition to known signals, the spectrum of the post‐mortem PNDI‐EG cathode exhibited five sharp additional signals. At first glance, these peaks appear to be similar to monomer signals, however, the FT‐IR spectrum of post‐mortem PNDI‐EG cathodes is nearly identical to that of the uncycled cathode. The origin of these ^13^C ssNMR peaks thus remains unclear. The signals assigned to the EO linker are largely retained after cycling in both networks.

In comparison to the as‐prepared and uncycled PMDI‐EG composite cathode, the IR spectra of fully discharged post‐mortem PMDI‐EG composite cathodes after prolonged cycling indicate the complete absence of the stretching and bending vibrations of the imide ring^[^ ^28^, ^34^, ^35^ ^]^ at ≈1000 and 720 cm^−1^ (Figure 3c). Furthermore, the two IR bands at 1770 and 1720 cm^−1^ representing the carbonyl symmetric and anti‐symmetric stretching modes, shift to ≈1550 cm^−1^ in the post‐mortem cells. The computed DFT IR spectra of pristine MDI (Figure 3e) and NDI (Figure S5, Supporting Information) describe well the two characteristic symmetric and anti‐symmetric ─C═O stretching modes, matching the experimental data. Upon lithiation (Figure S5, Supporting Information), calculated spectra show a remarkable red shift of the ─C═O stretching bands, that gradually decrease their vibrational frequencies from 1Li to 2Li species.^[^ ^28^ ^]^ At the same time, an enhancement of the C═C stretching band (1500–1600 cm^−1^) related to the change in electron delocalization of the conjugated core is observed.

Provided that the redox process is reversible and degradation does not occur, the IR spectra of post‐mortem cathodes in the fully discharged state should be similar to those of as‐prepared cathodes, as well as the computed, DFT‐based IR spectra of pristine MDI and NDI. This is true for PNDI‐EG composite cathodes, where the IR spectra of as‐prepared and post‐mortem cathodes are almost identical (Figure 3d), well showing the presence of the IR active bands related to the carbonyl groups and the imide ring. In contrast, post‐mortem PMDI‐EG composite cathodes appeared to have undergone irreversible changes and do not contain the imide rings. The carbonyl group stretching bands were shifted to a lower frequency. As the post‐mortem cathodes are in a fully discharged state, this red shift is not the result of lithiation but rather of other structural changes such as ring opening. To further investigate the origin of MDI degradation, the IR spectra of two possible imide ring‐opened products, i.e., acid amides, were simulated using DFT calculations (Figure 3e). While the stretching vibration of the imide ring is not present in the open structures, clear markers of the acid amides are N─H stretching vibrations at ≈3370 cm^−1^ and N─H bending and C─N stretching vibrations at ≈1570 cm^−1^. Notably, their intensities increase from the asymmetrically to the symmetrically ring‐opened structure. A closer comparison of the simulated IR spectra of symmetrically ring‐opened MDI and the PMDI‐2EG post‐mortem cathode suggests ring‐opened amides in the post‐mortem cathode as possible structures (Figure 3f).

The electrochemical reduction of phthalimide in dimethylformamide with tetraethylammonium perchlorate as supporting electrolyte was shown to lead to imide ring‐opening via protonation of the phthalimide dianion to form 3‐hydroxyphthalimidine, which is in equilibrium with the ring‐opened aldehyde.^[^ ^26^ ^]^ Cyclic imides can also undergo reversible ring opening under basic conditions facilitated by a nucleophilic catalyst.^[^ ^36^ ^]^ The potentially larger ring strain of the five‐membered MDI ring would result in a faster ring opening and would render MDI more susceptible to side reactions.^[^ ^37^, ^38^ ^]^ With pure lithium as the anode, trace impurities such as moisture or oxygen could create a basic medium in the cell. Under such conditions and with other nucleophilic species like the TFSI anion, although weak, or decomposition products such as LiF, the herein observed irreversible imide ring opening and degradation of PMDI‐EG‐based cathodes can be explained.

## 3 Conclusion

We have designed, synthesized and characterized networks of pyromellitic diimide (MDI) and naphthalene diimide (NDI). The networks PMDI‐EG and PNDI‐EG were synthesized via cationic polymerization of bis‐vinyl ethylene glycol‐functionalized MDI‐EG‐vin and NDI‐EG‐vin monomers, which were obtained in a single step under Mitsunobu conditions. The cross‐linked structures of PMDI‐EG and PNDI‐EG exclude a potential dissolution in the electrolyte as a source of electrode degradation. The electrochemical performance of PMDI‐EG and PNDI‐EG used as active cathode material in lithium‐ion battery half‐cells was markedly different. While in the initial charge–discharge cycles, both PMDI‐EG and PNDI‐EG composite cathodes delivered ≈90% of their theoretical capacity, the specific capacity of PMDI‐EG cathodes rapidly decreased upon prolonged cycling, maintaining only 9% capacity after 100 cycles. In contrast, PNDI‐EG‐based cathodes retained 96% capacity after 100 cycles and 86% capacity after 1000 cycles. Furthermore, the rate capability of PMDI‐EG cathodes was significantly inferior compared to PNDI‐EG. From a combination of solid‐state NMR spectroscopy, FT‐IR spectroscopy and DFT simulations, we concluded that PNDI‐EG‐based cathodes were rather stable during cycling while PMDI‐EG cathodes were susceptible to imide ring opening, leading to a rapid loss of capacity. The smaller ring size of the five‐membered MDI imide with a likely larger ring strain may be a contributing factor enabling ring opening of the imide upon side reactions with nucleophiles in the electrolyte.

## 4 Experimental Section

### Materials

Pyromellitic acid diimide (PMI‐H, 97%), naphthalene‐1,4,5,8‐tetracarboxylic dianhydride (NDA, > 95%), diethylene glycol monovinyl ether (stabilized with KOH, > 96%), triphenylphosphine (PPh_3_, 99%), diethyl azodicarboxylate (DEAD, 40% in toluene), SnCl_4_ (99%), Bu_4_NCl (98%), and NH_4_OH (25% aqueous solution) were purchased and used without further purification. 1,4,5,8‐Naphthalenetetracarboxylic Diimide (NDI‐H) was synthesized according to literature^[^ ^39^ ^]^ reacting NDA with NH_4_OH (25% aqueous solution) under argon.

### Instrument and Characterization

*Solid‐state nuclear magnetic resonance spectroscopy (ssNMR)* was performed at 9.4 T on a Bruker Avance 400 spectrometer equipped with double‐tuned probes capable of MAS (magic angle spinning). The finely powdered samples were packed into 3.2 mm rotors (OD) made of zirconium oxide spinning at 15 kHz. For ^13^C‐{^1^H}‐CP‐MAS NMR spectra, a cross polarization (CP) technique with contact time of 2 ms to enhance sensitivity, a recycle delay of 6 s, and ^1^H decoupling during acquisition using a TPPM (two pulse phase modulation) pulse sequence were applied. The spectra were referenced to tetramethyl silane (TMS) using tetrakis(trimethylsilyl)silane (TTSS) as a secondary standard (3.55 ppm for ^13^C and 0.27 ppm for ^1^H). *Solution NMR spectra* were collected using Bruker Avance Neo 600 FT spectrometer (600 MHz) in CD_2_Cl_2_ and referenced via solvent signals and a TMS standard. All NMR spectra were acquired at room temperature (25 °C).

*Differential scanning calorimetry (DSC)* was recorded under a continuous flow of nitrogen using a DSC 1 system from Mettler Toledo Analytical in combination with the STAR software using standard aluminum crucibles. The scan rate was 10 °C min^−1^ for all samples.

*Thermogravimetric analysis (TGA)* was performed in Netzsch Libra TG 209 F1 equipment in the temperature range of 30–600 °C with a heating rate of 10 °C min^−1^ under a continuous flow of argon.

*Elemental analysis* was performed with Vario MICRO cube (CHNS‐Analysis, Elementar Analysensysteme GmbH). 2–3 mg of samples were weighted in tin boat. The samples were then burn up at 1150 °C in the combustion tube in an oxygen‐helium gas mixture atmosphere. The reduction occurs in the reduction tube, filled with copper wires. The elements C, H, N develop gases CO_2_, H_2_O, N_2_, NO, NO_2_. These gases are separated on the U‐shaped separation columns, which are filled with special adsorber. The detection of the gases occurs with the thermal conductivity detector. For the calibration and quality control of the elemental analysis device, acetanilide was used as the standard.

*Fourier‐Transform Infrared (FT‐IR)* spectra were collected on an Alpha II FT‐IR spectrometer from Bruker at ambient conditions.

### Electrochemical Characterization

All characterizations including CV, charge–discharge cycling, rate‐capability tests were recorded using BioLogic BCS‐810 battery testing system at ambient conditions.

### Synthesis of Monomers NDI‐EG‐Vin via Mitsunobu Reaction^[^ ^40^ ^]^

To a 100 mL two‐neck‐round‐bottom (TNRB) flask NDI‐H (0.90 g, 3.38 mmol, 1.0 eq) was added. Under argon 40 mL anhydrous THF was added to the flask and then stirred that led to a dispersion. Afterward diethylene glycol monovinyl ether (1.8 mL, 16.91 mL, 5.0 eq) was added and then the mixture was cooled to 0 °C with an ice bath. At 0 °C PPh3 (2.48 g, 9.47 mmol, 2.8 eq) and DEAD (2.82 mL, 9.47 mmol, 2.8 mL) were added. The whole was warmed to rt and then refluxed at 85 °C for 48 hrs. The solvents were evaporated and the remaining oily crude product was first filtered through a short silica plug with ≈200 mL DCM: methanol (40: 1, V: V) as eluent. After evaporating the solvents, the yellow solid was crystalized with 150 mL of methanol: DCM (100:2, V: V), filtered and dried at 45 °C under vacuum to give 1.3 g pale yellow crystals (77%).

^1^H NMR (Figure S6, Supporting Information) (600 MHz, CD_2_Cl_2_ (5.31), *δ*): 8.73 (s, 4H, Ar H), Hb: 6.33 (dd, ^3^ *J*(*Z*) = 6.77 Hz, ^3^ *J*(*E*) = 14.30 Hz, 2H, ‐CH═CH_2_), 4.41 (t, ^3^ *J* = 5.99 Hz, 4H, ‐N‐CH_2_‐), 4.07 (dd, ^2^ *J* = 1.89 Hz, ^3^ *J*(*E*) = 14.30 Hz, 2H; ‐CH═CH_2_), 3.85 (dd, ^2^ *J* = 1.95 Hz, ^3^ *J*(*Z*) = 6.78 Hz, 2H; ‐CH═CH_2_), 3.81 (t, ^3^ *J* = 5.98 Hz, 4H; ‐N‐CH_2_‐CH_2_‐O‐), 3.75 (m, 4H; ‐CH_2_‐CH_2_‐O‐ CH═CH_2_), 3.71 (m, 4H; ‐CH_2_‐CH_2_‐O‐CH═CH_2_); ^13^C NMR (Figure S7, Supporting Information) (150 MHz, CD_2_Cl_2_ (53.84 ppm), *δ*): 163.32 (C═O), 152.11 (OCH═CH_2_), 131.23 (Aryl‐CH), 127.20 (Aryl‐C‐(C)_3_), 127.05 (Aryl‐C‐(C═O)(CH)), 86.73 (‐O‐CH═CH_2_), 69.66 (‐O‐CH_2_‐CH_2_‐O‐CH═), 68.19 (N‐CH_2_‐CH_2_‐O‐), 67.95 (‐O‐CH_2_‐CH_2_‐O‐CH═), 40.04 (N‐CH_2_‐CH_2_‐O‐); Anal. calcd. for C_26_H_26_N_2_O_8_: C 63.15, H 5.30, N 5.67; found: C 63.17, H 5.30, N 5.81.

### Synthesis of Monomers MDI‐EG‐Vin via Mitsunobu Reaction

MDI‐EG‐vin was synthesized and purified using the same procedures as for PNDI‐EG. PMI‐EG was obtained as white crystals in 71% yield.

^1^H NMR (Figure S8, Supporting Information) (600 MHz, CD_2_Cl_2_ (5.31), *δ*): 8.23 (s, 2H, Ar H), 6.35 (dd, ^3^ *J* (*Z*) = 6.76 Hz, ^3^ *J* (*E*) = 14.29 Hz, 2H; ‐CH═CH_2_), 4.09 (dd, ^2^ *J* = 2.00 Hz, ^3^ *J* (*E*) = 14.35 Hz, 2H; ‐CH═CH_2_), 3.92 (t, ^3^ *J* = 5.65 Hz, 4H; ‐N‐CH_2_‐), 3.89 (dd, ^2^ *J* = 2.00 Hz, ^3^ *J* (*Z*) = 6.76 Hz, 2H; ‐CH═CH_2_), 3.75 (t, ^3^ *J* = 5.45 Hz, 4H; ‐N‐CH_2_‐CH_2_‐O‐), 3.73 (m, 4H; ‐CH_2_‐CH_2_‐O‐CH═CH_2_),

3.66 (m, 4H; ‐CH_2_‐CH_2_‐O‐CH═CH_2_); ^13^C NMR (Figure S9, Supporting Information) (150 MHz, CD_2_Cl_2_ (53.84 ppm), δ): 166.61 (C═O), 152.09 (‐CH═CH_2_), 137.63 (Aryl‐C‐(C)_3_), 118.37 (‐CH(Aryl)), 86.82 (‐CH═CH_2_), 69.60 (O‐CH_2_‐CH_2_‐O‐CH═CH_2_), 68.04 (N‐CH_2_‐CH_2_‐O), 67.90 (O‐CH_2_‐CH_2_‐O‐ CH═CH_2_), 38.44 (N‐CH_2_‐CH_2_‐O); Anal. calcd for C_22_H_24_N_2_O_8_: C 59.46, H 5.44, N 6.30; found: C 59.55, H 5.48, N 6.32.

### Synthesis of PNDI‐EG via Cationic Polymerization

To a 50 mL Schlenk flask with a magnetic stirring bar NDI‐EG (395.6 mg, 0.8 mmol, 1.0 eq) and Bu_4_NCl (4.45 mg, 0.016 mmol, 0.02 eq) were added. The flask was subjected to three cycles of argon‐vacuum‐argon before adding 20 mL anhydrous THF to dissolve the monomer. The mixture was degassed by Freeze‐pump‐thaw degassing method for three cycles. After that at rt SnCl_4_ (41.64 mg, 0.16 mmol, 0.2 eq) was injected and the flask was placed into an oil bath at 40 °C and the mixture was stirred for 24 h. Upon cooling to rt the reaction mixture was poured into 200 mL methanol, filtered, the solids were washed with acetone and Soxhlet extracted with acetone for one day before drying at 50 °C under vacuum. The cross‐linked polymer PNDI‐EG (276 mg, 70%) was obtained as light brown solid (Scheme 1). Anal. calcd for C_26_H_30_N_2_O_8_: C 62.64, H 6.07, N 5.62; found: C 62.75, H 5.44, N 5.38.

### Synthesis of PMDI‐EG via Cationic Polymerization

PMDI‐EG was synthesized following the same procedure as PNDI‐EG and was obtained as light brown solid in 58% yield (Scheme 1). Anal. calcd for C_22_H_28_N_2_O_8_: C 58.92, H 6.29, N 6.25; found: C 59.73, H 5.63, N 5.89.

### General Procedure for PNDI‐EG/PMDI‐EG Composite Cathode Preparation

General Procedure for Cathode Preparation. The cross‐linked polymer PNDI‐EG or PPMI‐EG (80 mg, 80 wt.%) and MWCNT (20 mg, 20 wt.%) were mixed well in a pestle and mortar. Then, to the polymer‐MWCNT mixture (100 mg, 95 wt.%) PVDF was added (5 wt.%, 5.263 mg) and the whole dissolved in 1.3 mL N‐methyl‐2‐pyrrolidinone (NMP). The cathode slurry was stirred at room temperature for 24 h and coated onto a nickel mesh substrate (0.5 mm thickness, ø 14 mm), which was first dried at rt for 24 h followed by another 20 h at 65 °C. The final composite cathode has PNDI‐EG/PPMI‐EG (76 wt.%): MWCNT (19 wt.%): PVDF (5 wt.%) composition and a typical mass loading of active polymer of 2.5 mg.cm^−2^ unless otherwise stated.

*General procedure for coin half‐cell assembly*: 2032‐type coin cells were assembled in an Ar‐filled glove box (Braun, O_2_ and H_2_O < 0.6 ppm) with PNDI‐EG/PMDI‐EG composite coated onto nickel mesh as cathodes and lithium metal as anodes with copper foils as the current collector. Prior to closing the cell, the separators were soaked with 50 µL of 2 m bis‐(trifluoromethane)‐sulfonimide lithium salt (LiTFSI) and 0.25 m lithium nitrate (LiNO_3_) mixture in 1:1 (V: V) 1, 2‐dimethoxy‐ethane (DME):1, 3‐dioxolane (DOL) as electrolyte mixture. The electrolyte to PNDI‐EG/PPMI‐EG ratio was kept in the range of 12−15 mL·g^−1^. The cells were left in the bench at rt for 20 h before testing.

### Quantum Chemical Calculations

NDI and PMI molecular structures, in their neutral, mono‐ and di‐lithiated states, were initially optimized at the tight binding DFT‐based semi‐empirical level (GFN2‐xTB), with the code xtb (v.6.7.1).^[^ ^41^, ^42^ ^]^ Each structure was further optimized and refined at the DFT level by using the B3LYP‐D3 exchange‐correlation functional and the triple‐zeta correlation consistent basis set (cc‐pVTZ). DFT calculations were carried out with the Gaussian16 (v.C.01) code.^[^ ^43^ ^]^

## Conflict of Interest

The authors declare no conflict of interest.

## Supporting information



Supporting Information

## Data Availability

The data that support the findings of this study are available from the corresponding author upon reasonable request.

## References

[1] P. Poizot , J. Gaubicher , S. Renault , L. Dubois , Y. Liang , Y. Yao , Chem. Rev. 2020, 120, 6490. 32207919 10.1021/acs.chemrev.9b00482

[2] T. B. Schon , B. T. McAllister , P.‐F. Li , D. S. Seferos , Chem. Soc. Rev. 2016, 45, 6345. 27273252 10.1039/c6cs00173d

[3] Y. Lu , J. Chen , Nat. Rev. Chem. 2020, 4, 127. 37128020 10.1038/s41570-020-0160-9

[4] B. Esser , F. Dolhem , M. Becuwe , P. Poizot , A. Vlad , D. Brandell , J. Power Sources 2021, 482, 228814.

[5] D. Mecerreyes , N. Casado , I. Villaluenga , M. Forsyth , Macromolecules 2024, 57, 3013. 38616814 10.1021/acs.macromol.3c01971 PMC11008248

[6] K. Qin , J. Huang , K. Holguin , C. Luo , Energy Environ. Sci. 2020, 13, 3950.

[7] Q. Zhao , Y. Lu , J. Chen , Adv. Energy Mater. 2017, 7, 1601792.

[8] B. Pan , J. Huang , Z. Feng , L. Zeng , M. He , L. Zhang , J. T. Vaughey , M. J. Bedzyk , P. Fenter , Z. Zhang , A. K. Burrell , C. Liao , Adv. Energy Mater. 2016, 6, 1600140.

[9] D. J. Kim , D.‐J. Yoo , M. T. Otley , A. Prokofjevs , C. Pezzato , M. Owczarek , S. J. Lee , J. W. Choi , J. F. Stoddart , Nat. Energy 2019, 4, 51.

[10] H. Cui , L. Ma , Z. Huang , Z. Chen , C. Zhi , SmartMat 2022, 3, 565.

[11] B. Häupler , A. Wild , U. S. Schubert , Adv. Energy Mater. 2015, 5, 1402034.

[12] S. Y. An , T. B. Schon , B. T. McAllister , D. S. Seferos , EcoMat 2020, 2, e12055.

[13] L. Zhu , G. Ding , L. Xie , X. Cao , J. Liu , X. Lei , J. Ma , Chem. Mater. 2019, 31, 8582.

[14] C. Thalacker , C. Röger , F. Würthner , J. Org. Chem. 2006, 71, 8098. 17025298 10.1021/jo0612269

[15] S. Hameury , S. Kunz , M. Sommer , ACS Omega 2017, 2, 2483. 31457594 10.1021/acsomega.7b00420 PMC6640996

[16] N. Zindy , J. T. Blaskovits , C. Beaumont , J. Michaud‐Valcourt , H. Saneifar , P. A. Johnson , D. Bélanger , M. Leclerc , Chem. Mater. 2018, 30, 6821.

[17] S. V. Bhosale , M. A. Kobaisi , R. W. Jadhav , P. P. Morajkar , L. A. Jones , S. George , Chem. Soc. Rev. 2021, 50, 9845. 34308940 10.1039/d0cs00239a

[18] M. Sommer , J. Mater. Chem. C 2014, 2, 3088.

[19] G. S. Vadehra , R. P. Maloney , M. A. Garcia‐Garibay , B. Dunn , Chem. Mater. 2014, 26, 7151.

[20] X. Guo , A. Facchetti , T. J. Marks , Chem. Rev. 2014, 114, 8943. 25181005 10.1021/cr500225d

[21] K. T. Tan , S. Ghosh , Z. Wang , F. Wen , D. Rodríguez‐San‐Miguel , J. Feng , N. Huang , W. Wang , F. Zamora , X. Feng , A. Thomas , D. Jiang , Nat Rev Methods Primers 2023, 3, 1.

[22] M. S. Lohse , T. Bein , Adv. Funct. Mater. 2018, 28, 1705553.

[23] J. Chen , S. Gu , R. Hao , K. Liu , Z. Wang , Z. Li , H. Yuan , H. Guo , K. Zhang , Z. Lu , ACS Appl. Mater. Interfaces 2022, 14, 44330. 36125517 10.1021/acsami.2c11138

[24] S. Renault , V. A. Mihali , K. Edström , D. Brandell , Electrochem. Commun. 2014, 45, 52.

[25] G. Hernández , N. Casado , R. Coste , D. Shanmukaraj , L. Rubatat , M. Armand , D. Mecerreyes , RSC Adv. 2015, 5, 17096.

[26] D. W. Leedy , D. L. Muck , J. Am. Chem. Soc. 1971, 93, 4264.

[27] G. Hernández , M. Salsamendi , S. M. Morozova , E. I. Lozinskaya , S. Devaraj , Y. S. Vygodskii , A. S. Shaplov , D. Mecerreyes , J. Polym. Sci., Part A: Polym. Chem. 2018, 56, 714.

[28] Y. Shi , H. Tang , S. Jiang , L. V. Kayser , M. Li , F. Liu , F. Ji , D. J. Lipomi , S. P. Ong , Z. Chen , Chem. Mater. 2018, 30, 3508.

[29] R. E. Oueslati , B. Jismy , B. Flamme , N. Leclerc , F. Ghamouss , M. Abarbri , J. Mater. Chem. A 2024, 12, 15866.

[30] R. Matsidik , K. Skudler , S. de Kock , A. Seifert , M. Müller , M. Walter , S. Choudhury , M. Sommer , ACS Appl. Energy Mater. 2023, 6, 9466.

[31] S. de Kock , K. Skudler , R. Matsidik , M. Sommer , M. Müller , M. Walter , Phys. Chem. Chem. Phys. 2023, 25, 20395. 37465922 10.1039/d3cp02285d

[32] S. U. Sharma , Y.‐L. Chang , S. V. Chaganti , Y. W. More , J.‐T. Lee , ACS Appl. Energy Mater. 2022, 5, 7550.

[33] C. Sandford , M. A. Edwards , K. J. Klunder , D. P. Hickey , M. Li , K. Barman , M. S. Sigman , H. S. White , S. D. Minteer , Chem. Sci. 2019, 10, 6404. 31367303 10.1039/c9sc01545k PMC6615219

[34] M. Lv , F. Zhang , Y. Wu , M. Chen , C. Yao , J. Nan , D. Shu , R. Zeng , H. Zeng , S.‐L. Chou , Sci. Rep. 2016, 6, 23515. 27064938 10.1038/srep23515 PMC4827395

[35] S.‐H. Hsiao , C.‐P. Yang , C.‐W. Chen , G.‐S. Liou , J. Polym. Res. 2005, 12, 289.

[36] M. B. Kim , D. W. Dixon , J. Phys. Org. Chem. 2008, 21, 731.

[37] S. Huijser , E. HosseiniNejad , R. Sablong , C. de Jong , C. E. Koning , R. Duchateau , Macromolecules 2011, 44, 1132.

[38] E. Hosseini Nejad , C. G. W. van Melis , T. J. Vermeer , C. E. Koning , R. Duchateau , Macromolecules 2012, 45, 1770.

[39] S.‐M. Chen , L.‐M. Chang , X.‐K. Yang , T. Luo , H. Xu , Z.‐G. Gu , J. Zhang , ACS Appl. Mater. Interfaces 2019, 11, 31421. 31389682 10.1021/acsami.9b11872

[40] M. Nakano , M. Sawamoto , M. Yuki , K. Takimiya , Org. Lett. 2016, 18, 3770. 27428656 10.1021/acs.orglett.6b01785

[41] S. Grimme , S. Ehrlich , L. Goerigk , J. Comput. Chem. 2011, 32, 1456. 21370243 10.1002/jcc.21759

[42] C. Bannwarth , E. Caldeweyher , S. Ehlert , A. Hansen , P. Pracht , J. Seibert , S. Spicher , S. Grimme , WIREs Computational Mol. Sci. 2021, 11, e1493.

[43] M. J. Frisch , et al., Gaussian 16, Revision C.01, Gaussian, Inc, Wallingford CT, 2016.

