# Supplementary material for: Naphthalene Diimide and Pyromellitic Diimide Networks as Cathode Materials in Lithium‐ion Batteries: on the Instability of Pyromellitic Diimide
Source: Macromol Rapid Commun. 2025 Jan 13;46(6):2401121. doi: 10.1002/marc.202401121 (PMC11925328; doi:10.1002/marc.202401121)
Supplement: Supplementary file 1 — Supporting Information [file MARC-46-2401121-s001.docx]

Supporting Information

Naphthalene Diimide and Pyromellitic Diimide Networks as Cathode Materials in Lithium-ion Batteries: On the Instability of Pyromellitic Diimide

Rukiya Matsidik, Daniele Fazzi, Andreas Seifert, and Michael Sommer*


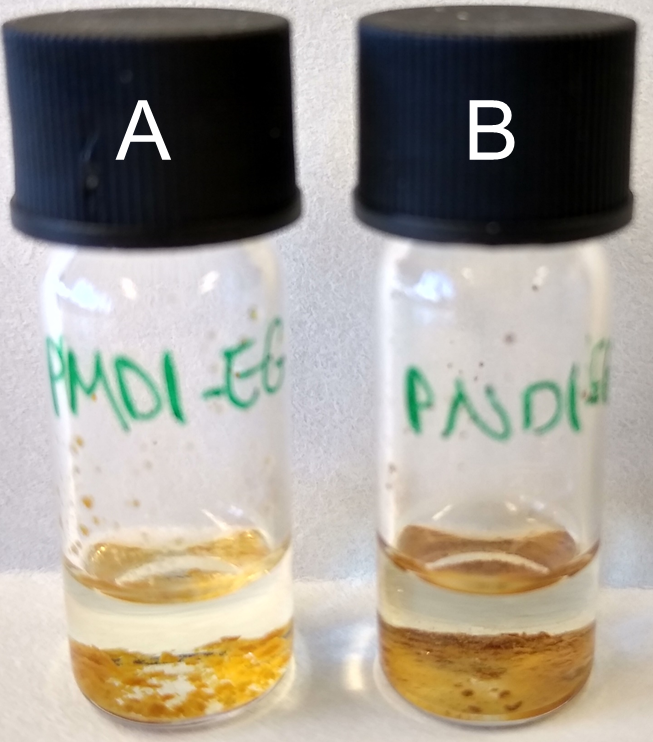


**Figure S1**. Pictures of PMDI-EG (A) and PNDI-EG soaked in the electrolyte mixture DOL: DME (v: v, 1: 1) with a concentration of ca. 2 mg/mL for one week.

**
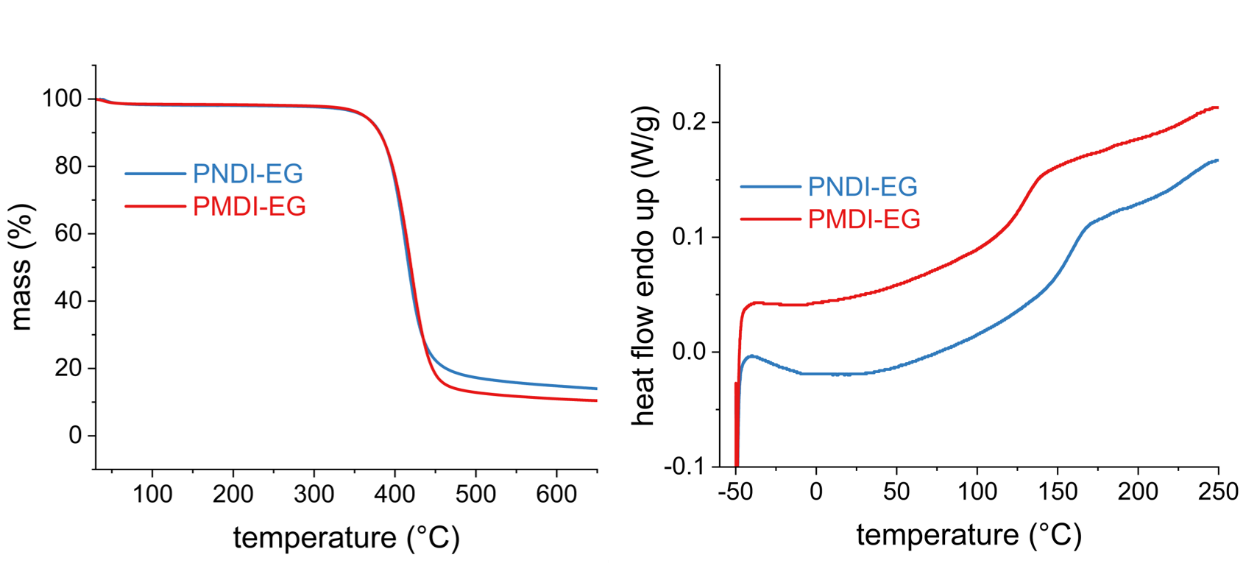
**

**Figure S2.** TGA (right) and DSC (left) curves of PNDI-EG (blue) and PMDI-EG (red).


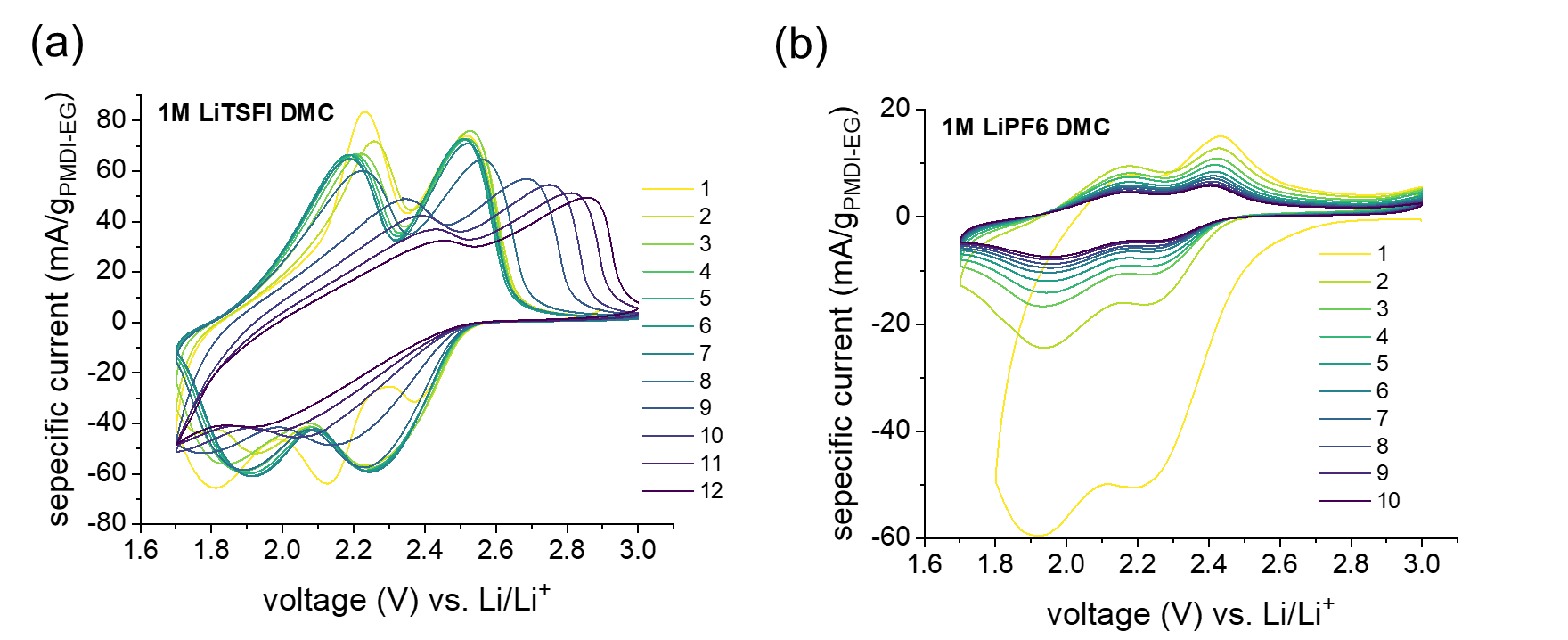


**Figure S3**. CV curves of PMDI-EG composite cathodes with a scan rate of 0.1 mV/s in dimethyl carbonate (DMC) in two different lithium salt electrolytes: (a) 1 M LiTSFI and (b) 1 M lithium hexafluorophosphate (LiPF6).





**Figure S4**. Long-term cycling of PNDI-EG (blue) and PMDI-EG (red) composite cathode/Li half cells.

**
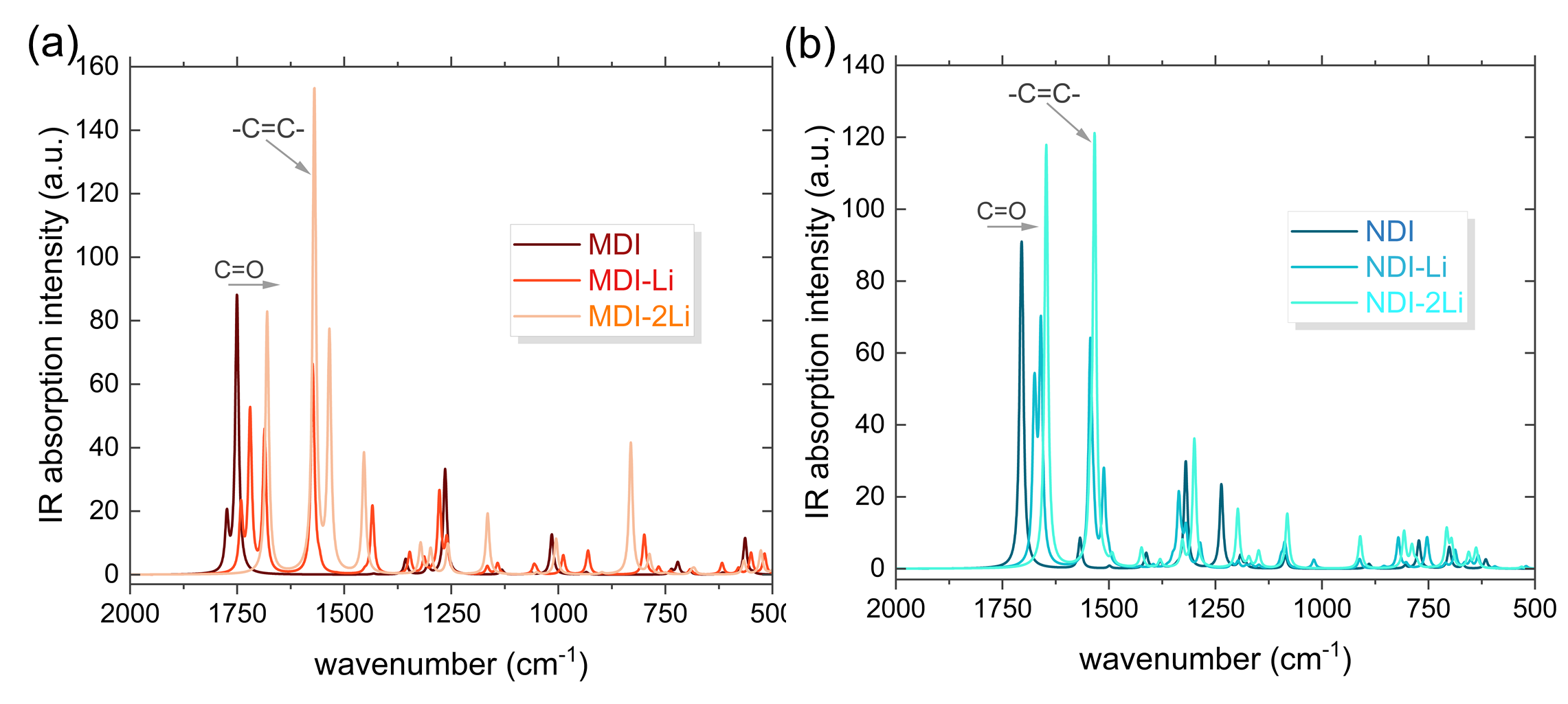
**

**Figure S5**. DFT calculated IR spectra of natural, one and two lithium added species of MDI (a) and NDI (b).

**Table S1.** Calculated (at B3LYP-D3/cc-pVTZ level) molecular orbital energy levels of neutral, one time and two times lithiated species of MDI and DNI.

| **molecule** | **HOMO (eV)** | **LUMO (eV)** |
| --- | --- | --- |
| NDI | -7.463 | -3.810 |
| NDI-Li | -4.289 (alpha)  -6.170 (beta) | -2.446 (alpha)  -2.923 (beta) |
| NDI-2Li | -3.678 | -1.762 |
| MDI | -8.024 | -3.537 |
| MDI-Li | -8.024 (alpha)  -4.134 (beta) | -2.346 (alpha)  -2.496 (beta) |
| MDI-2Li | -3.079 | -1.726 |


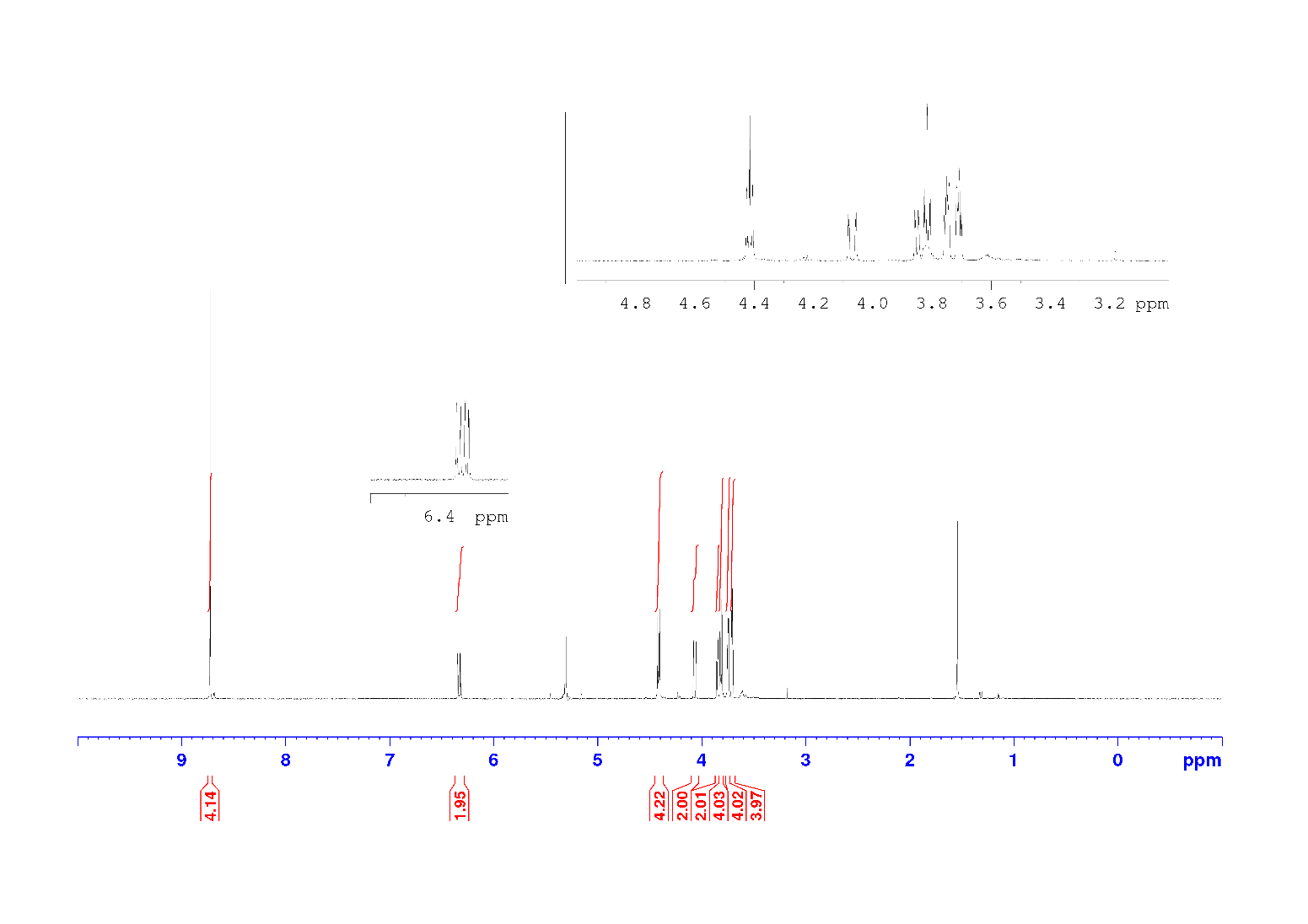


**Figure S6**. ^1^H NMR (600 MHz, CD_2_Cl_2_) spectrum of NDI-EG-vin.


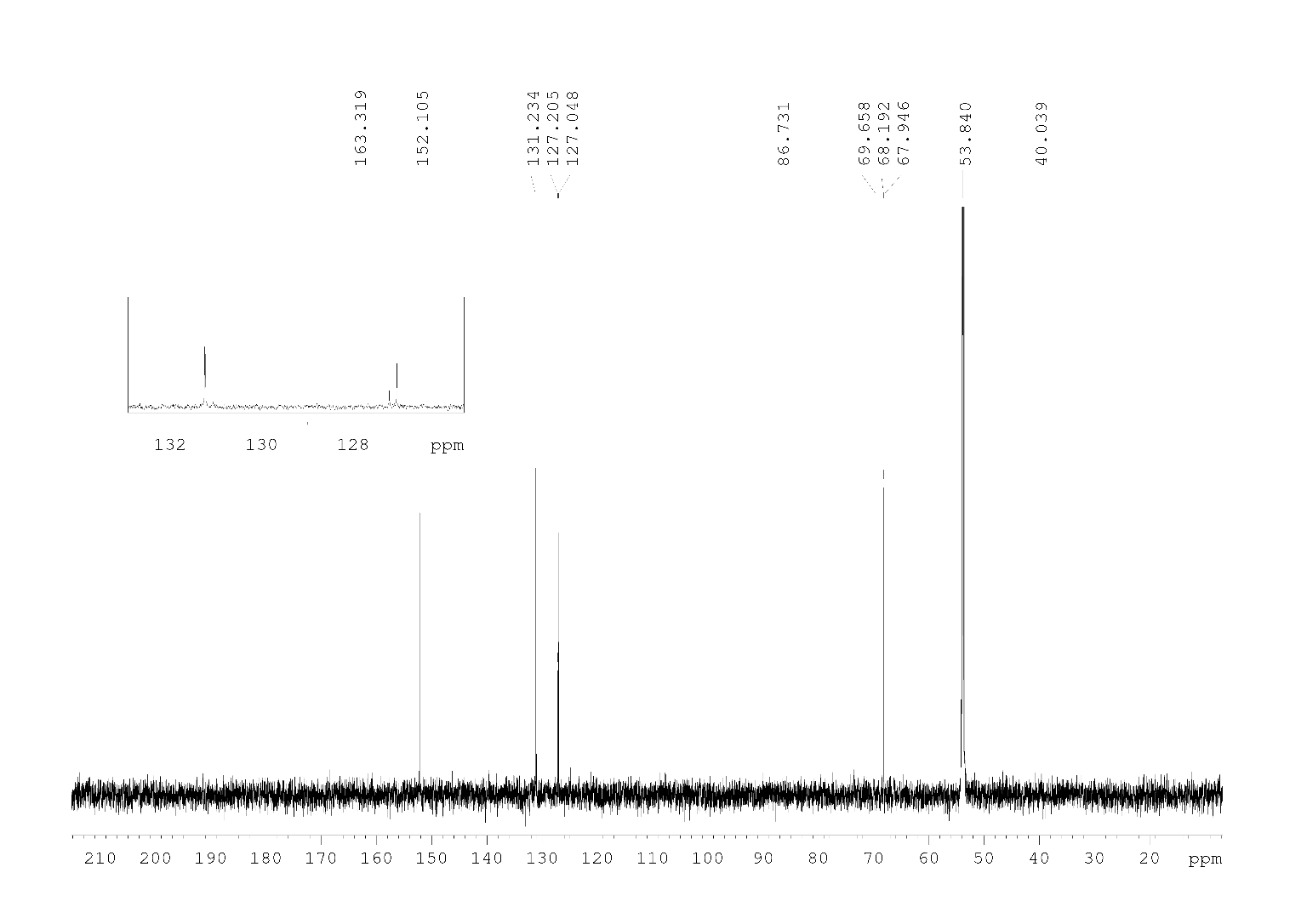


**Figure S7**. ^13^C NMR (150 MHz, CD_2_Cl_2_) spectrum of NDI-EG-vin.


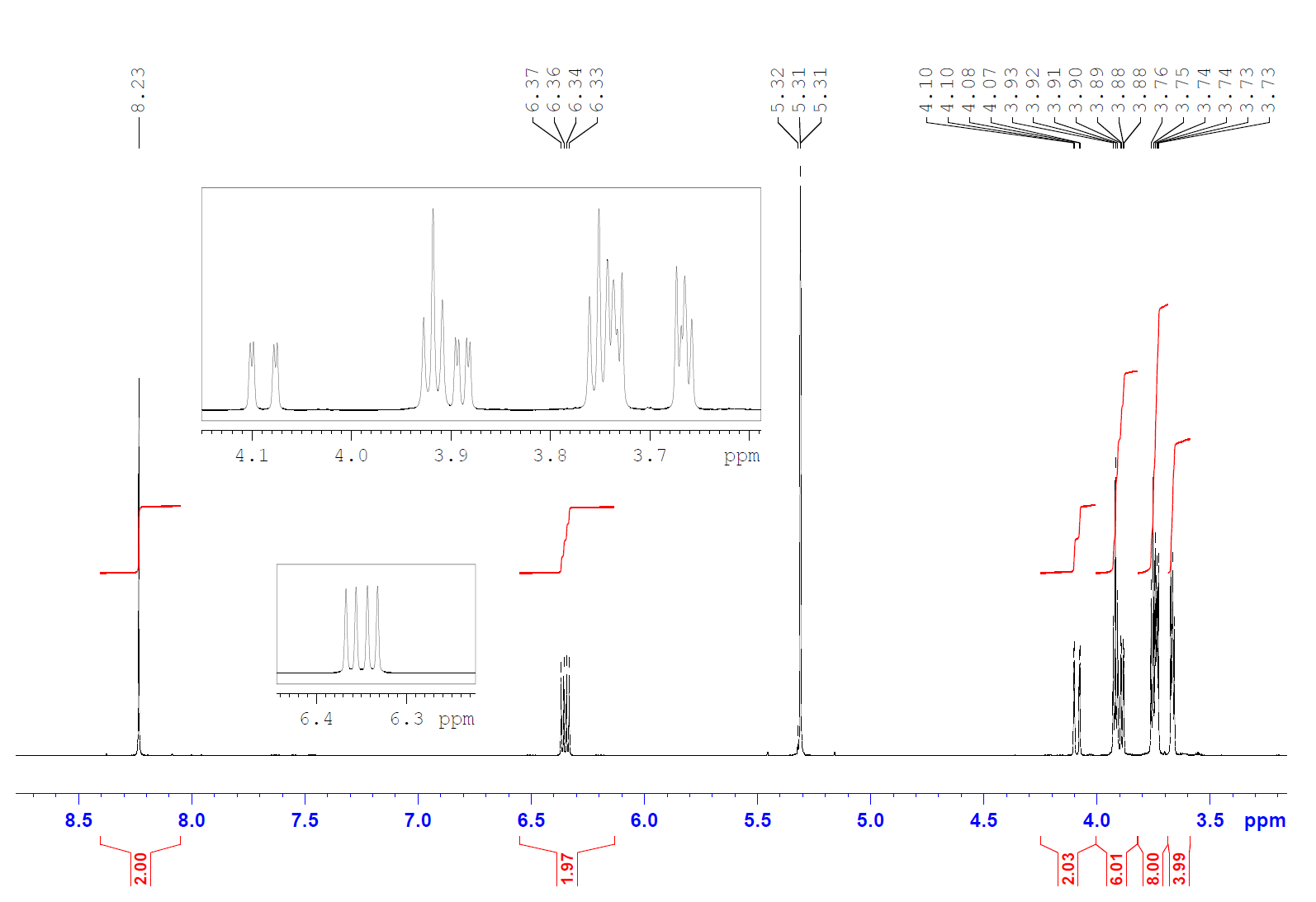


**Figure S8**. ^1^H NMR (600 MHz, CD_2_Cl_2_) spectrum of MDI-EG-vin.

**
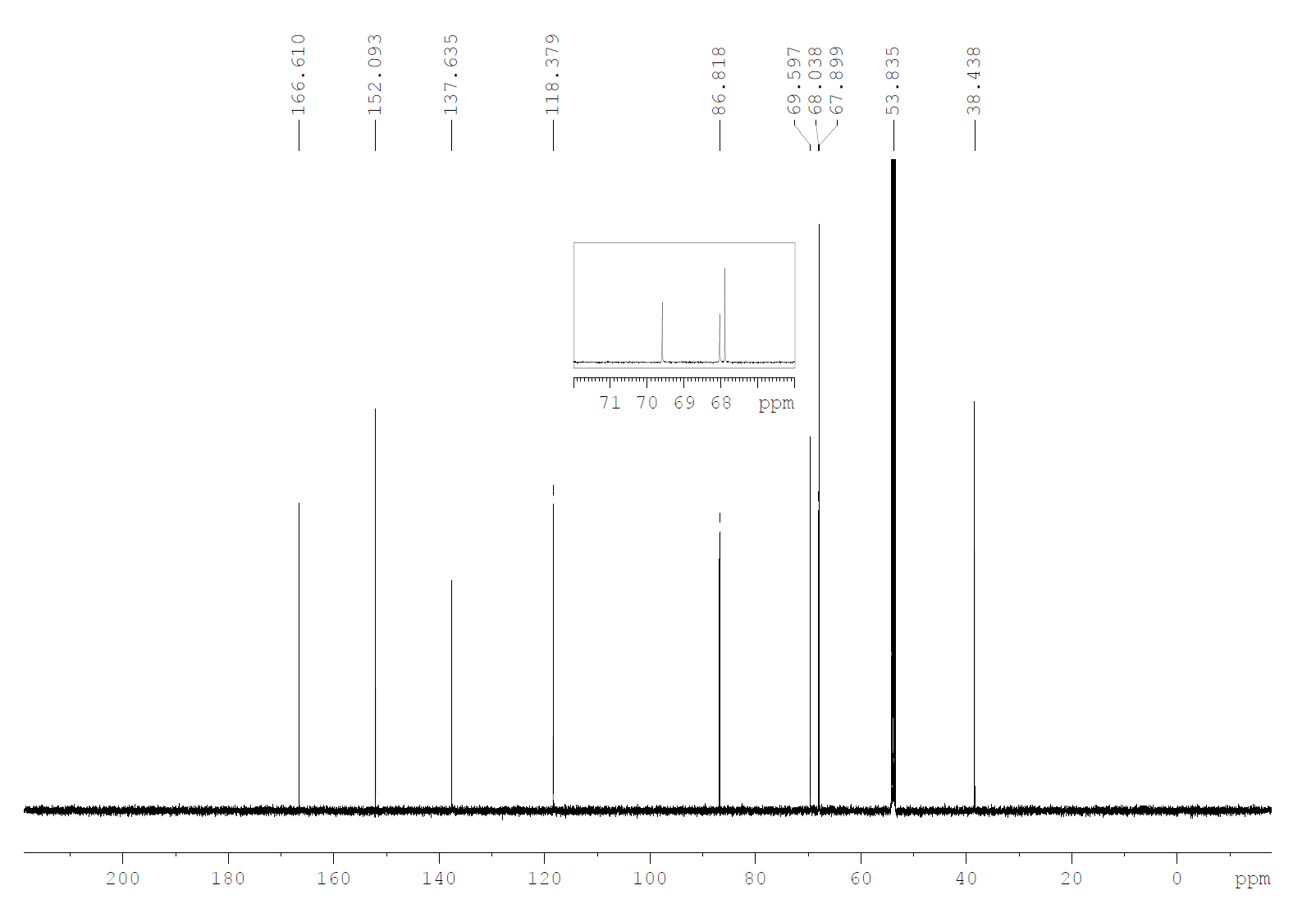
**

**Figure S9**. ^13^C NMR (150 MHz, CD_2_Cl_2_) spectrum of MDI-EG-vin.
